# The challenges in protecting public health interests in multisectoral governance in the context of small island developing states: the case of tobacco control in Fiji and Vanuatu

**DOI:** 10.1186/s12992-023-00931-y

**Published:** 2023-04-28

**Authors:** Dori Patay, Ashley Schram, Sharon Friel

**Affiliations:** 1 School of Regulation and Global Governance, College of Asia and the Pacific, The Australian National University, Canberra, Australia; 2 The George Institute for Global Health, Sydney, Australia; 3 Menzies Centre for Health Policy and Economy, Sydney School of Public Health, The University of Sydney, Camperdown, NSW 2006 Australia

**Keywords:** Small island developing states, Pacific, Commercial determinants of health, Noncommunicable diseases, Multisectoral policy, Multisectoral governance, Whole-of-government, Tobacco control, Fiji, Vanuatu

## Abstract

**Background:**

The commercial determinants of health (CDoH) drive the rise of NCDs globally, and their regulation requires multisectoral governance. Despite existing recommendations to strengthen institutional structures, protecting public health interests can be challenging amidst industry interference and conflicting policy priorities, particularly in low and middle-income countries (LMICs) where the need for rapid economic development is pronounced. Small island developing states (SIDS) face even more challenges in regulating CDoH because their unique socioeconomic, political, and geographic vulnerabilities may weaken institutional conditions that could aid health sector actors in protecting health interests. This study aims to explore the institutional conditions that shape health sector actors’ capability to protect public health interests in tobacco governance in Fiji and Vanuatu.

**Methods:**

We employed a qualitative, exploratory case study design. We applied the administrative process theory to inform data collection and analysis. Seventy interviews were completed in Fiji and Vanuatu from 2018 to 2019.

**Results:**

The findings show that the protection of health interests in tobacco governance were not supported by the institutional conditions in Fiji and Vanuatu. While the policy processes formally ensured a level playing field between actors, policies were often developed through informal mechanisms, and the safeguards to protect public interests from vested private interests were not implemented adequately. SIDS vulnerabilities and weak regulation of political parties contributed to the politicisation of government in both states, resulting in high-level government officials’ questionable commitment to protect public health interests. The system of checks and balances usually embedded into democratic governments appeared to be muted, and policymakers had limited bureaucratic autonomy to elevate health interests in multisectoral policymaking amidst high-level government officials’ frequent rotation. Finally, capacity constraints aggravated by SIDS vulnerabilities negatively impacted health sector actors' capability to analyse policy alternatives.

**Conclusions:**

Health sector actors in Fiji and Vanuatu were not supported by institutional conditions that could help them protect public health interests in multisectoral governance to regulate CDoH originating from the tobacco industry. Institutional conditions in these states were shaped by SIDS vulnerabilities but could be improved by targeted capacity building, governance and political system strengthening.

## Background

The noncommunicable diseases (NCDs) epidemic is a major burden for societies and health systems globally [1]. The increased consumption of harmful commodities, including tobacco, alcohol and ultra-processed foods, drives the global rise of NCDs [2]. The need to regulate the commercial determinants of health (CDoH) – the systems, practices and pathways through which commercial actors increase the availability, affordability, accessibility and demand for harmful commodities [3] – has been recognised by public health scholars [4, 5]. A multisectoral approach to govern CDoH is needed, as the production, distribution, trade and consumption aspects of harmful commodities are regulated by a diverse range of government sectors besides the health sector, including industry, trade and finance [6, 7].

Democratic governments tend to provide multiple opportunities for actors to advance their interests in policymaking. This can enable health sector actors to influence non-health sector policies, such as trade, industry, agriculture, or economy, to optimise their public health impact. However, these processes also provide a chance for commercial actors to formally influence policymakers [8], where they aim to ensure a policy and regulatory environment that favours their interests (so-called industry interference) and maximises profits [9]. This has been recognised as a major challenge for public health, as these actors’ profit-oriented interests often conflict with public health interests.

Tobacco governance serves as a great example where health sector actors are challenged to protect public health interests. In this study, tobacco governance is defined as the formal and informal mechanisms and conditions that shape how tobacco is produced, manufactured, traded and consumed. Multiple actors participate in tobacco governance: for example, government agencies that regulate tobacco within policy sectors of health, agriculture, trade, industry, or education; industry actors who profit from producing, manufacturing and trading tobacco; and civil society actors who may advocate for heightened tobacco control. The ways in which the tobacco industry engages and influences governments are the most documented among the harmful commodity industries [3], and the responsibilities of governments to protect public health policymaking from industry interests is the most binding compared to alcohol or ultra-processed foods because of the Framework Convention for Tobacco Control (FCTC) [10]. Despite the significant progress in tobacco control globally, high smoking prevalence continues to be a major health issue in low- and middle-income countries (LMICs), where health sector actors often struggle to protect public health interests in tobacco governance [11].

To protect health sector actors from tobacco industry interference and to help elevate health interests among the interests of other government sectors, recommendations have been developed to establish certain institutional structures. For example, whole-of government mechanisms provide platforms where government agencies can discuss how their policies can be aligned [12, 13], and the establishment of [12, 13] terms of engagement with the tobacco industry (Article 5.3 of FCTC) can create safeguards against industry interference [10]. Despite these recommended institutional approaches, commercial interests often overcome health interests in tobacco governance [14]. In LMICs, economic development is often the primary concern of governments [4, 15, 16], and the tobacco industry’s arguments on the economic benefits provided by their activities are often well-received [14, 17]. This is a significant issue as LMICs experience 77% of the global mortality from NCDs [1], and while tobacco use is predicted to be responsible for the death of 8 million people annually by 2030, 80% of these deaths will happen in LMICs [18].

Small island developing states (SIDS) are a unique group of LMICs situated in the Caribbean, the Pacific, Africa, the Indian Ocean, the Mediterranean and the South China Sea [19]. SIDS have unique socioeconomic, political, and geographic conditions – often called SIDS vulnerabilities – that make their development particularly challenging [19]. These SIDS vulnerabilities that set them apart from other LMICs, include the small size of their land, population and economies; geographic isolation (from other countries and between islands of the same state); governments that are small in size (fitting to the scale of the population) and with limited human, technical and financial resources; low administrative capacity; infrastructural challenges of travel and supply of goods and services, and their dependence on the policies and conduct of larger economies in their region [20].

Pacific small island developing states (PSIDS) declared an NCD crisis in 2011 [21]. Despite the majority of PSIDS adopting the Tobacco Free Pacific 2025 Goal [22], the rate of current smokers among males reaches as high as 54–74% in some states – in contrast to the global average of 22% [11, 23–26]. Previous research has shown that PSIDS face challenges in implementing multisectoral NCD prevention policies [27, 28], and that their SIDS vulnerabilities potentially aggravate government fragmentation and their susceptibility to tobacco industry influence [16, 29–31].

Understanding what institutional conditions make health sector actors able to protect policymaking from the corporate interests of tobacco and other harmful commodities is vital for tackling the NCD crisis because it can help identify ways to strengthen governance structures accordingly. Institutional conditions in this study are defined as the structures and attributes of governance in a given setting; for example, these can include structures, processes, capacities or policymakers’ characteristics [32]. The lessons learned about how some of the smallest and least powerful countries can overcome the vested interests of some of the most powerful companies may benefit other, larger countries with more resources to tackle CDoH. Yet, the literature on public health policymaking in PSIDS provides limited analysis of institutional conditions that affect multisectoral governance [33–36], and the scholarship on tobacco governance is PSIDS is scarce [30, 37]. Therefore, this paper aims to explore the institutional conditions in two PSIDS – Fiji and Vanuatu – that shape health sector actors’ capability to protect public health interests in tobacco governance. This study was conducted as part of a larger research project investigating the ways interests, ideas, and institutions shape tobacco governance in Fiji and Vanuatu [38].

## Methods

We applied a theory-informed, exploratory research design, relying on qualitative interviewee data.

### Theoretical perspective

Data collection and analysis were guided by Croley’s administrative process theory [32], which arose from new institutionalist scholarship that interprets governance through the operation of institutions [39]. New institutionalist theories can be divided into four categories. Rational choice institutionalism argues that institutions are “structures of incentives” within which individuals act based on their calculated interests [40]. Historical institutionalism describes the development of institutions based on the “logic of path-dependence”, meaning that the ways structures are formed follow certain patterns and practices [40]. Sociological institutionalism suggests that individuals within institutions act based on the “logic of appropriateness” – they follow the pressures of society in their actions [40]. Discursive institutionalism argues that individuals follow the norms, beliefs, and ideas dominant in their institution [40]. Croley’s administrative process theory belongs to the rational choice institutionalist theories, arguing that calculated interests drive decisions within institutions [32].

The administrative process theory claims that policymakers can ensure that governance is not dominated by vested interests if five institutional conditions are in place: (i) policymaking processes ensure a level playing field between actors; (ii) extra-legislative mechanisms, such as judicial reviews and presidential oversight, help maintain policy maker’s autonomy from politics; (iii) policymakers are committed to protecting public interests; (iv) policymakers have the capacity and capability to conduct a careful analysis of policy alternatives; and (iv) policymakers have considerable bureaucratic autonomy (both from politicians and other dominant fractions of the government) [32].

The administrative process theory offers an explanation of what conditions help government agencies resist vested interests, and it was successfully used in a prior tobacco control study [32]. Thus, this theory was deemed suitable for the purpose of this paper to investigate health sector actors’ capability to protect public health interests in tobacco governance. Five theoretical constructs were drawn from the administrative process theory to inform the data collection and analysis for this study, presented in Table 1.

**Table 1:** The claims of the administrative process theory, theoretical constructs and reporting themes

**The claims of the administrative process theory** (the institutional conditions needed for public policies to not be dominated by vested interests)	**Theoretical constructs** (used in data collection and analysis to investigate health sector actors' ability to protect public health interests in tobacco governance)	**Themes** (subsections of the Results section)
Policymaking processes ensure a level playing field between actors	Administrative procedures balancing out interest group influences	Policymaking procedures: (i) the policy and legislative process, (ii) terms of engagement with the tobacco industry
Extra-legislative mechanisms, such as judicial reviews and presidential oversight, help maintain policy maker’s autonomy from politics	Institutional environment	Checks and balances
The policymakers are committed to protect public interests	Policymakers commitment to protect public interests	Policymakers commitment to protecting public interests
Policymakers have the capacity and capability to conduct a careful analysis of policy alternatives	Cost–benefit analysis of policy alternatives	Capability to analyse policy alternatives for public interest
Policymakers have considerable bureaucratic autonomy (both from politicians and other dominant fractions of the government)	Bureaucratic autonomy	Bureaucratic autonomy

### Study design, data collection and analysis

#### Study design

An exploratory, qualitative case study approach with a two-case design was applied that incorporated within-case analysis and cross-case synthesis [41–43]. The two-case design raises the analytical strength of the research because the results arising from the two cases can be contrasted, improving the accuracy and generalisability of the findings and reducing uncertainty [42]. This design allows the exploration of the conditions surrounding the intersectoral governance mechanisms of tobacco control in two countries with similar contexts [44]. The within-case analysis is defined as “*the in-depth exploration of a single case as a stand-alone entity*” [42]; it allows the deep analysis of the institutional conditions characterising tobacco governance in both contexts [42]. A cross-case synthesis – combining evidence from the two cases [44] – was applied to aggregate the findings of each within-case analysis. This allows a case-based approach rather than a variable-based approach because it synthesises the results without reducing the data to variables, thus keeping their holistic features [44]. This approach enables the synthesis of within-case patterns while keeping the integrity of each case [44]. The selected study design enables analysis of the findings from the two cases within a single analytical framework, and it is particularly efficient for in-depth analysis of the conditions behind governance mechanisms [45]; thus, it suits this exploratory study well. Prior tobacco control and food policy studies successfully employed a similar approach in PSIDS [34, 36, 37, 46, 47].

#### Case study selection

As the purpose of this study was to learn about what makes health sector actors capable of protecting tobacco governance from vested industry interests, we aimed to identify case study countries where public health interests were safeguarded in tobacco governance (i.e., multisectoral tobacco control measures were put in place despite the opposition of tobacco industry interests). Therefore, first, we selected PSIDS with recent progress in implementing FCTC measures that regulate the tobacco industry, using MPOWER reports^1^ [48]. Then, we identified two PSIDS where tobacco industry interests were prevalent (and thus, health sector actors were likely needed to protect tobacco governance from industry influence): Fiji was chosen as a country where commercial tobacco farming and manufacturing were detected based on tobacco-related exports [49, 50]; Vanuatu was selected as a country without an established tobacco industry but with interest in investing in these sectors, according to recent news articles that were identified through a Google search.

Fiji has a population of approximately 900,000, scattered over 110 islands [51]. Since regaining independence from the British Empire in 1970, its politics has been characterised by a series of coups, with the latest in 2009 [52]. In 2014, the democratic government was reinstated [52]; however, the same political elite remained in power until December 2022 [53]. Tobacco farming was introduced to Fiji during colonial times; however, the tobacco industry was officially established only in 1973, first dominate by two and later one local industry actor. In 2000 British American Tobacco (BAT) bought this local tobacco company (562). Since then, BAT has controlled the entire supply chain of tobacco in Fiji. It is the second largest multinational tobacco corporation in the world after Philip Morris International, with the total revenue of 31.88 billion US$ in 2021 [54]. Tobacco continues to be considered a highly profitable cash-crop among farmers, and BAT has a close relationship with the political elite [16, 55]. Yet, in 2005, the Fijian government ratified FCTC, and the sale, advertising and promotion, labelling, tar and nicotine content, and public consumption of tobacco have been regulated by the Tobacco Control Act 2010 and the Tobacco Control Regulations 2012 [56, 57]. The prevalence of current smokers among males and females was 47.0% and 14.3%, respectively, at the time of the last survey (2011) [58].

Vanuatu counts a population of approximately 410,000 and 65 habituated islands [51]. Central Intelligence Agency [51] Since regaining its independence in 1980, Vanuatu has had frequently changing governments [51]. Tobacco is grown on a very small scale; however, the local and Australian media reveal the efforts the tobacco industry has been making to establish itself in the country since 2012 [59]. Until recently, the industry hadn’t been successful, but in 2019 the construction of the first tobacco factory began in Port Vila (although later stopped by the Water Department) [60, 61]. The country ratified FCTC in 2005, and the Tobacco Control Act 2008 and Tobacco Control Regulations 2013 regulate tobacco sales, advertising, promotion, and marketing; labelling; reporting and limitations of contents; and smoke-free places [62, 63]. The prevalence of current smokers among males and females was 46.0% and 4.0%, respectively, at the time of the last survey (2011) [64].

#### Data collection

Data was collected through in-depth interviews between April 2018 and August 2019. As tobacco governance is inherently multisectoral and shaped by a range of government, civil society and private actors, a purposive and snowball selection process was applied that targeted participants from government agencies in health, agriculture, trade, industry, finance, and education; civil society organisations; local academic institutions; development partners (intergovernmental and regional organisations, and governmental agencies of donor countries); and the tobacco industry. Most interviews were conducted in person and a few over Skype, with an average duration of 60 min (between 35–80 min). The interviews were semi-structured, with open-ended questions that were informed by the theoretical constructs. For example, questions included: “*Are there any terms of agreement or safeguards in place to protect government officials from tobacco industry interference?*” or “*How much bureaucratic autonomy do the Department of Public Health policy officers have?*” Interviews were audio-recorded after informed consent; in those cases when the consent did not include audio-recoding, the researcher took written notes. Follow-up interviews were conducted in those cases when the data required further clarification.

#### Data analysis

Interviews were transcribed and cleaned by the lead author, then thematically coded against the theoretical constructs listed in Table 1 with a deductive approach in NVivo. To strengthen the quality of the data collection and analysis, source triangulation was applied: similar questions were asked from multiple participants and follow-up interviews were conducted to clarify and validate findings as necessary [65]. In addition, the analysis was discussed with the co-authors, and the results were validated with selected participants.

## Results

Forty-two interviews were conducted in Fiji and 28 in Vanuatu; 21 invitees (including the tobacco industry) declined participation. The number of interviews based on actor type and country is provided in Table 2. In the following, the results are presented by themes and are summarised in Table 3.

**Table 2:** The distribution of interviews

		**Fiji**	**Vanuatu**
**Type of actor**	Government agencies	25	21
	Civil society organisations & academic institutions	3	1
	Development partners (intergovernmental and regional organisations, and governmental agencies of donor countries)	14	6
**Policy sector**	Health	21	10
	Trade/Industry	3	5
	Agriculture	1	1
	Finance/Economy	4	1
	Education	2	2
	Foreign Affairs	2	1
	Multisectoral	9	8

**Table 3:** The summary of the findings by themes derived from the administrative process theory

Themes		Key findings
Policymaking procedures	(i) Policy and legislative process	Several formal opportunities for intersectoral negotiations in Fiji and Vanuatu
		Gap between rules and actual policymaking
		Centralised decision-making in Fiji
		Inadequate consultations
		Limited capacity or intention for stakeholder involvement
		Limited stakeholder capacity for meaningful contribution
	(ii) Terms of engagement with the tobacco industry	Lack of official terms of engagement
		Regular interaction and inadequate transparency measures
		Limited screening for individual conflicts of interest
		Limited oversight from third parties
		No awareness raising or lobby transparency measures on tobacco industry interference
Checks and balances		Weak Parliament and limited oversight
		Limited judicial and executive oversight
Policymakers commitment to protect public interests		High-level government officials were perceived to have low commitment to protecting public health interests
		Clientelism and patronage as possible drivers of low commitment to protecting public health interests
		Weak political part regulation contributing low commitment to protecting public health interests
		Mid- and low-level government officials were more likely to be committed to protecting public health interests
Capability to analyse policy alternatives for public interest		Limited human capacity
		Limited technical capacity
		Low financial capacity
		Issues in performance management and accountability
Bureaucratic autonomy		Muted layers of decision-making and accountability in Fiji
		The role of political culture in policy makers’ limited proactivity in Fiji
		Frequent changes in strategic direction
		Public service reforms but with similar issues in Fiji

### Policymaking procedures

According to the administrative process theory, democratic policy processes should ensure a level playing field and multiple opportunities for actors to influence policy development [32], and Article 5.3 of FCTC requires the establishment of terms of engagement to protect public health policies from tobacco industry influence [10]. The analysis revealed that although certain safeguards were embedded into policymaking procedures in Fiji and Vanuatu, compliance with these procedures were problematic in both countries.

#### Policy and legislative process

Formally, the policy and legislative process offered several opportunities for intersectoral negotiations in Fiji and Vanuatu. The data revealed at least four opportunities when health sector actors could elevate public health interests: two were identified during policy development and another two in the legislative process. First, during the policy development, consultations had to be conducted with the public and relevant actors. A government official explained this as follows: “*The government will never accept anything if it didn’t go through consultations with public, NGOs, government departments, sellers, producers, importers, everybody*” (F03, Government).

Second, inter-ministerial discussions were to be held in the Cabinet (which in Vanuatu was preceded by an additional lower-level inter-ministerial meeting). If the policy was embedded into legislation, the third opportunity presented itself before the bill was debated in Parliament: a Parliamentary Standing Committee conducted an evidence review and consultations. Finally, actors could directly lobby Members of Parliament (MPs) who voted on the bill once the Standing Committee report was tabled.

While these opportunities to elevate public health interests were encouraging for tobacco control, multiple participants highlighted the gap between the formal rules and the actual ways policies were developed:

*We have a lot of policies and whether decision makers use them to implement or make decisions is another question. (V25, Government)*

*There was one example of a health-related legislation […] The amendment was proposed, drafted, all in the absence of MoH [Ministry of Health]. Virtually a night before it was proposed to Parliament, someone gave a copy to MoH [Ministry of Health]: “just let you know, this is what’s going to happen tomorrow.” (F34, Development partner)*

The analysis suggested that several conditions were likely behind the poor compliance with the policy and legislative processes in Fiji and Vanuatu. First, the data showed that decision-making was heavily centralised in Fiji. Participants indicated that the political elite, who gained power in the 2008 coup, held significant decision-making authority in the government. Centralisation of authority within the government was quite apparent. For example, the Attorney-General (AG) served as the Minister for Economy, Civil Service, Communications, and Housing and Community Development, while the PM was also the Minister for Sugar Industry, Foreign Affairs, iTaukei Affairs, and Forestry [66]. The participants described the AG’s decision-making authority as follows:

*It will be really just decided by the AG. (F34, Development partner)*

*Either the PM, or the Minister for Economy. They are the ones who will make the final decision in terms of the policy formulation. (F21, Government)*

Second, the data indicated that in both countries, consultations were often conducted inefficiently, involved only a few actors, were held too late in the policy process, or the collected input was not incorporated into the policy. As a government official recalled a consultation, “*just a few people came in […] But it does not matter if people come or not, we just had to prove that it was done*.” (F04, Government) The data suggested that actor engagement was poor, not because of capacity or organisational issues, but because the agency did not have any real intention of considering other opinions and interests. In Fiji, this was explained as a remnant of the military regime: “*The government is not used to a process of consultations before they make decisions. […] [It] is making some attempts to change, but I think in some areas they do not want to consult*” (F34, Development partner). In Vanuatu, there was often no intention to implement the policy paper itself, just to support funding requests towards development partners. As a government official recalled:

*It's just kind of ‘all right, let's get the policy document in place. Then we'll go on with the work we were doing anyway’, rather than identifying it as an opportunity to put in place some real change. […] It was like, we just need to write something, and the more people we involve, the more time it will take, and we do not have time. (V15, Government)*

Third, other participants recalled that when consultations or intersectoral meetings were held, they did not necessarily bring the desired input, either because relevant actors did not attend or not contribute meaningfully to the discussion:

*We took [the policy draft] to a stakeholder consultation after it was written. They were generally like, 'yeah, looks good'. Again, I still do not think people in that room read it. […] There were about 40 actors from across the public sector, NGOs and to the private sector. (V15, Government)*

*When MoH [Ministry of Health] calls Finance, Trade, Planning to come for a meeting, these guys do not even come or they send a small officer who cannot take any decision. (F28, Development partner)*

This analysis revealed that the policy and legislative processes in Fiji and Vanuatu had safeguards embedded to protect public health interests; however, these processes were often not followed.

#### Terms of engagement with the tobacco industry

The analysis showed a lack of official terms of engagement with the tobacco industry in Fiji and Vanuatu. According to the MANA dashboard, Vanuatu had no tobacco industry interference policies in place, while Fiji was in the process of developing one [27]. However, the interviews did not reflect any progress on this front. The lack of terms of engagement was particularly problematic in light of the close relationship between the tobacco industry and government agencies in Fiji and Vanuatu [16, 38], and because in both countries, the administrative procedures – for obtaining and maintaining registration and licensing of the tobacco industry – required the Ministry of Health (MoH) to interact with industry representatives. Government officials described these processes as follows:

*They [tobacco related businesses] renew their licences through the ministry [MoH], so if they have any issues with the packaging or that kind of things, they have to liaise with the ministry. (F06, Government)*

*Firstly, they [tobacco industry] have to go to the Vanuatu Investment Promotion Authority to apply for a permit. […] The Department of Industry comes in when it comes to processing tobacco, but for the planting they need to talk to the Ministry of Agriculture. They also need to talk with the MoH to see their regulations. (V16, Government)*

Without implemented transparency measures, this provided an opportunity for the tobacco industry to influence high-level policymakers. For example, in 2018, tobacco industry representatives met with the Minister for Health to secure MoH endorsement for establishing the first tobacco factory in Vanuatu. No transparency measures were in place, and the industry representatives left with a signed supporting letter [paraphrased, V17]. In 2020, the same precedent was repeated [61, 67].

The data showed that limited mechanisms were in place to screen individual conflicts of interest. MoH in Fiji had a procedure in place to screen prospective administrators for individual conflicts of interest related to tobacco. A government official explained that “*they do not employ people coming from pro-tobacco sector. They do a very thorough check on the applicants.”* (F06, Government).

However, such a mechanism was not applied in the Ministry of Economy or Agriculture, as the hiring of ex-BAT employees to high-level positions indicated [68, 69]. For example, a member of Parliament (MP) stated that “*the Head of Procurement in the Ministry of Economy is someone who has come from British American Tobacco*” [69]. In Vanuatu, the interviews reflected no process in place to filter out applicants with conflicts of interest.

The analysis showed limited oversight from third parties in tobacco governance. Oversight by civil society organisations (CSOs), development partners or the public may limit vested interest influence over policymakers. However, no transparency and accountability measures were in place in Fiji and Vanuatu, and no tobacco industry watchdog or tobacco control-related CSO operated in these countries. A participant explained that “*not only in tobacco, but they do not have any CSOs which are looking into NCDs at all*” (F27, Development partner).

Participants explained that neither country had any activities on awareness raising of industry interference practices, and no lobby transparency measures were in place either [paraphrased, F26]. A government official stated the following: “*The only monitoring happens by the NCD officer at WHO [in Suva]. She Googles it and then lets MoH know. But in the Ministry [MoH] nobody does it*” (F06, Government).

These findings showed that the policymaking procedures in place in Fiji and Vanuatu contain limited safeguards to protect public health interests; however, compliance with the procedures have been weak in both states. The system of checks and balances that is embedded in democratic systems should ensure that policymaking procedures are followed, and public interests are not overtaken by narrow, vested interests. The next section presents the findings how Parliamentary and judicial oversight in Fiji and Vanuatu provides these additional safeguards.

### Checks and balances

The analysis indicated the weakness of Parliament in both countries to provide oversight over policymaking. In Fiji, development partners have been providing capacity building to MPs since democratic institutions were reinstated in 2014 [70]. Although Vanuatu has had democratic institutions since its independence in 1980, its Parliamentary mechanisms were still under development: not only do the MPs needed to be trained on the legislative procedures, but the technical and procedural capacity of the Standing Committees needed improving [70]. Consequently, the Parliament in Vanuatu was considered weak, and participants stated that the government (the ruling party) controls it:

*No one Is really knowledgeable in the Parliament at the moment. […] About the autonomy of Parliament, there is a huge lack to that extent in Vanuatu. Government is completely controlling the Parliament. […] There is no Parliamentary oversight. (V11, Development partner)*

In Fiji, strict confidentiality rules protected policymakers from needing to disclose information to other government agencies or the Parliament, limiting Parliamentary oversight. As a government official explained:

*Whatever decisions do not reach the Parliament from the Cabinet, it is mostly because it is confidential. […] Cabinet information is only released after prior approval from the PM and the Secretary of the Cabinet. (F07, Government)*

The analysis showed that judicial and executive oversight were limited in both Fiji and Vanuatu. In both countries, judicial oversight over the policy and legislative process was practised only when Constitutional rights are affected; the courts did not check whether the policy process was followed correctly. A government official explained that “*if to be a watchdog, to make sure that all the government functions are operating, no, the court does not do that*” (V31, Government).

The Supreme Court in Vanuatu had been noted to be independent of the Parliament; participants explained that it has regularly prosecuted a high number of MPs for corruption [71, 72] and that the judiciary in Vanuatu tended to control corruption among MPs but did not exercise much oversight over the administration. The same proactivity was not so visible in the Fijian judiciary; a participant suggested that possibly the AG had influence over the courts [paraphrased, F35]. The executive oversight by the President had not shown any relevance in either state.

The findings showed that the system of checks and balances embedded into democratic governance appeared to be muted in Fiji and Vanuatu: the Parliament, the Judiciary and the President in both countries seemed to have limited control over policymakers to ensure that the formal policymaking procedures are followed and that public interests are protected against vested interests. The next part of the analysis showed that this was a major issue for tobacco governance, because high-level government officials were not necessarily determined to protect public health interests.

### Government officials’ commitment to protecting public health interests

The analysis showed that high-level government officials engaged in tobacco governance were perceived to have a low commitment to protect public health interests in Fiji and Vanuatu. Overall, in Fiji and Vanuatu, participants stated that government officials at the highest levels were caught up in politics, which often had detrimental effects on their commitment to protecting public health interests in tobacco governance. Others suggested that pursuing short-term economic benefits served high-level government officials’ political interests more than long-term health benefits. As participants explained:

*People up the top there do not take [tobacco control] seriously enough. Who is on top? PS [Permanent Secretary] and Minister for Health. (F06, Government)*

*Now only the “technicians” want to move things, but there is no real drive from the top. The top is focused on winning the elections and building economic growth. (F28, Development partner)*

The analysis indicated that clientelism and patronage were possible drivers of government officials’ low commitment to protecting public health interests in tobacco governance. Clientelism is the “*proffering of material goods in return for electoral support”* [73], and patronage means the “*exchange of a public sector job for political support”* [73]. In Vanuatu, SIDS vulnerabilities, such as the country's small population and land size, geographical isolation of islands resulting in infrastructural challenges, developing economy and the weakness of the government contributed to the limited reach and efficiency of public services. Consequently, communities perceived that they only benefit from politics if their representatives were elected to Parliament, a perception that provided a solid ground for clientelism. Government officials explained this as follows:

*Politics is localised. Every small place tries to choose someone from their area in the hope that that person will bring them something or they'll accept some form of bribe to vote for that person again because it's the only thing they see; they do not get much in services. (V13, Government)*

The data showed that weak political party regulation contributed to government officials’ low commitment to protecting public health interests in tobacco governance. This low commitment was also seen as being shaped by weak political party regulation, which contributed to corruption and frequent political power changes. The regulation of how political parties operate (e.g., who may establish a party and how, how it needs to be funded) was reported to be weak in Vanuatu. Consequently, and also fuelled by the geographically fragmented constituency, 24 parties were operating in 2014, with this number decreasing to 17 in 2016. To form a majority government, parties needed to form coalitions, and high-level government positions were often offered in exchange for support (i.e., patronage). As a participant stated:

*There are many different political parties. […] In Parliament you need to build a majority, so the main group in power will try to build a coalition, and they will just buy off the people: “Come, I will give you a minister post”. (V11, Development partner)*

Participants suggested that because of patronage, important positions in the government were given to people who were neither skilled nor had the experience for the job:

*I do not think there's anything that's in the decision-making in the government that is not politicised. Even hiring people, especially jobs that are high-level positions. It's not what you can bring to the table, but whom you know. (V27, Development partner)*

*The Ministers of Health over many years have been all sorts of people with no interest or knowledge. (V13, Civil society)*

In addition, and due to the limited political system regulation, political power structures frequently shifted in Vanuatu: the person of the Prime Minister (PM) was changed seven times between 2008 and 2012 and four times between 2012 and 2016. Between 2016 and 2020, the PM stayed in his role but had to overcome several impeachment attempts. As political alliances frequently shifted in Vanuatu, high-level government offices often changed hands; those occupying them were aware of the possibility of short-term duration and, thus, were likely to prioritise personal political interests instead of public interests. As a participant explained: “*One of the issues is that most of them only think about their political interests, parties’ interests, but not national interest*.” (V11, Development partner).

However, the data showed that mid-level MoH officials who stayed in one position for a significant length of time tended to be committed to protecting public health interests. While the Ministers in Fiji and Vanuatu and the Permanent Secretaries^2^ (PSs) in Fiji often changed several times a year, other lower executive and mid-level officers often served for a longer period. Moreover, the Department heads in both countries and the Director-General (DG) of MoH in Fiji were highly regarded for protecting public health interests. As a government official stated: “*The DG has been kind of steering a steady ship at times that has been captured by ministers that do not really understand health in any way*.” (V15, Government) The progress in tobacco control in both Fiji and Vanuatu was often explained by these mid-level government officials’ leadership.

The findings around government officials’ commitment to protecting public health interests showed that the clientelism and patronage in both countries – aggravated by SIDS vulnerabilities and weak regulation of the political space – often resulted in high-level MoH officials in power who were not necessarily motivated by the public but rather personal or political interests. However, mid-level government officials were more likely to be dedicated to protecting public health interests; the following two sections explain whether these officers’ departments had the necessary capacity and bureaucratic autonomy to do so.

### Capability to analyse policy alternatives for public interest

According to the administrative process theory, government agencies are able to conduct their own analysis to determine the most beneficial policy alternatives for the public and avoid excessive reliance on the information provided by particular actors (such as the tobacco industry) only if they have the adequate human, technical, financial and administrative capacity [32]. However, MoH in both Fiji and Vanuatu faced capacity issues common in SIDS.

The data indicated limited human capacity in tobacco governance in Fiji and Vanuatu. A participant explained that while in larger LMICs, often an entire unit is responsible for tobacco control, due to small populations, in SIDS, there is often one person dedicated to NCDs or tobacco control; thus, smoking is likely to receive less attention [paraphrased, F27]. This statement confirmed other government officials’ claim that tobacco control receives limited resources in Fiji and Vanuatu. There was one government official dedicated full-time to tobacco control policymaking and a number of environmental health officers worked on compliance; however, long-term vacancies in these positions were common. This was likely to have a detrimental effect on the agency's performance. For example, a government official stated: “*We have a very good policy environment in Vanuatu for public health. It’s just that there are limited human resources. It is difficult with the scattered islands*” (V30, Government).

The data also showed limited technical capacity in tobacco governance in both countries. Acquiring the necessary technical skills in areas related to tobacco governance was challenging in Fiji and Vanuatu due to the limited higher education courses available in local universities. Thus, finding policymakers with the required skills took a long time. As government officials explained:

*The Director of Planning just came in less than six months ago. Prior to that, that post has been vacant for the past three to four years. (V29, Government)*

*One person has negotiated agreements, but he now has left. So we don't have any trading negotiations now; we have no trade negotiators. (V25, Government)*

In addition, the low financial resources dedicated to NCDs were commonly seen as a barrier to strengthening tobacco control among government officials, as the following quote suggests:

*Even though the government declared an NCD crisis, not only in Vanuatu but in the Pacific, when it comes to sharing the funds, communicable diseases still receive more money. (V12, Government)*

Furthermore**,** participants expressed their opinion that performance management and accountability issues constrained administrative capacity within the government:

*I do not think we have enough good people in the right positions in government. The workplace culture in Vanuatu in the government is not very conducive to having performance determine your position. People can be underperforming, and it's never picked up, and it happens all the time. (V25, Government)*

These findings showed that SIDS vulnerabilities have a detrimental impact on health sector actors' capability to analyse policy alternatives for the public interest. Although human and financial resource problems are conditions commonly observed in LMICs, the data demonstrated that in Fiji and Vanuatu, such issues were aggravated by geographical isolation, the small size of the population and its economy, and the logistical and financial challenges of distributing services across several islands. However, besides capacity, government departments dedicated to protecting public health needed to have a necessary amount of bureaucratic autonomy to ensure that they can protect public health interests from other fragments of the government that might represent pro-tobacco interests. The following sections give an account of bureaucratic autonomy within the governments of Fiji and Vanuatu.

### Bureaucratic autonomy

The data showed muted layers of decision-making and accountability in Fiji. As described earlier, the AG and the PM held multiple important ministerial positions in Fiji. Moreover, the policy process was designed in a way that either the AG or the Minister for Economy needed to approve policies – who had been the same individual between 2014 and 2022. Additionally (or perhaps consequently), government agencies, in general, rarely initiated their ideas but waited for the AG and the PM to identify priorities and issue areas. A participant explained this as follows:

*In a lot of instances, ministries won’t actually provide or do any policy development […] Everything is really centrally controlled, even when it is related to individual actions of ministries, nobody takes any step forward unless it’s approved by him [AG]. (F34, Development partner)*

The analysis revealed that political culture played an important role in policymakers’ limited proactivity in Fiji. Although decision-making should be practised at each administrative level vertically during the development of policy, these layers of decision-making were often muted in MoH in Fiji. According to participants, the PS often held the most decision-making power within the ministry. Participants suggested that this had been complemented by the risk-aversion culture heavily present in the Fijian civil service: officials often tried to avoid making decisions not to make any mistakes that could result in losing their job [paraphrased, F34]. Given how often PSs changed in MoH – arguably, the person who made most decisions within the agency – this looked like a realistic fear: within MoH, there were four PSs between 2016 and 2018, which showed that keeping a PS position in this ministry was challenging. A participant suggested that the Public Service Commission (controlled by the AG and the PM) changed the PS of MoH as soon as enough time had passed for the individual to understand how the sector works because that was when they could start to have independent ideas [paraphrased, F02]. Thus, by avoiding the responsibility for decision-making, the necessary layers of accountability were also lacking. Consequently, government officials often lacked proactivity in policy development (F23, F34). For example, a participant stated:

*Ministers say that ‘nobody does anything until I ask them to do it’. But if anyone acts in a proactive way, they are not sure how they are going to be received, therefore, there is very little will from the public servants to be proactive. (F34, Development partner)*

According to government officials, the muted layers of decision-making and accountability contributed to the frequent changes in MOH’s strategic direction when the top leadership rotated in Fiji and Vanuatu:

*The expectations of our department change all the time; as the management changes, the action plan changes. (F09, Government)*

*We had a change of minister about five times while I was in MoH in those two years. Which is incredibly frustrating. […] When a new minister would come in, we'd see an overhaul of what we were doing. (V15, Government)*

However, government officials in Vanuatu suggested that department heads often retained a certain amount of bureaucratic autonomy.

*If the things that come from below their [Director of Public Health] level, they generally got a fair bit of autonomy to do what they like, but for things that come from above by the DG or the minister, they generally are a bit more propelled into doing it, whether they agree with it or not. (V15, Government)*

The Ministry of Civil Service recognised the nuisance of the lack of autonomy that resulted from the muted layers of decision-making and accountability; it had attempted to salvage this issue through a series of public service reforms between 2016 and 2022. However, the way the reforms were planned seemed to contradict their aim to give more responsibility to lower-level executives: participants explained that the PSs were responsible for planning and implementing such reforms in their respective ministries, and in MoH, even the Deputy Secretaries and Department Directors are excluded from this planning and decision-making process [paraphrased, F08, F14].

In summary, in Fiji, the centralisation of decision-making authority and the political culture left little bureaucratic autonomy in the hands of policymakers. These findings showed that even if policymakers are committed to protecting public health interests, they had little autonomy to ensure that health interests were elevated in tobacco governance. In Vanuatu, strategic priorities often changed due to the frequently changing political landscape, resulting in a heavy rotation of high-level MoH officials, which disrupted the strategic elevation of health interests in tobacco governance.

## Discussion

This paper explored the institutional conditions that shape health sector actors’ ability to protect public health interests in tobacco governance in Fiji and Vanuatu. Our study contributes to the commercial determinants of health, health governance and development literature by shedding light on the connection between SIDS vulnerabilities, political context, and government structures, rules, and accountability in tobacco governance in Fiji and Vanuatu. Previously no similar study has been published on PSIDS that provided a theory-informed, interdisciplinary analysis of the institutional conditions that shaped tobacco governance.

The findings showed that the protection of health interests in tobacco governance was not supported by the institutional conditions in Fiji and Vanuatu. While the policy processes formally ensured a level playing field between actors, policies were often developed through informal mechanisms, and the safeguards to protect public interests from vested private interests were not implemented adequately. The system of checks and balances embedded into democratic governance appeared to be muted in both countries, and SIDS vulnerabilities and weak regulation of the political parties contributed to the politicisation of government in both states, resulting in high-level government officials’ limited commitment to protect broad public interests (such as public health). Furthermore, capacity issues, aggravated by SIDS vulnerabilities, posed major limitations to health sector actors' capability to analyse policy alternatives, and policymakers had limited bureaucratic autonomy to elevate health interests in multisectoral governance amidst the frequent rotation of high-level government officials.

These findings showed that SIDS vulnerabilities had a major impact on the ways institutions were structured and operated in both states. In Vanuatu, clientelism and patronage were consequences of the country being scattered over multiple little islands, and due to the weak regulation of political parties, the political landscape rapidly changed in the country. As a result, individuals in executive government positions did not necessarily govern for the public interest but for their personal, political, or localised interests. Fiji, being a post-authoritarian state, had a centralised government system where the ministries were highly dependent on the AG's discretion, and since he often prioritised commercial interests, this constrained MoH from elevating health interests in tobacco governance. These political conditions negatively impacted the performance management and accountability mechanisms of government agencies, which were further burdened by the weak human and financial capacities common in SIDS. The consequences were twofold. Firstly, the rules of policymaking – which could ensure the protection of public health interests – were often not followed, and there were no terms of engagement implemented with the tobacco industry. Secondly, health sector actors had limited capacity and capability to make well-informed policy choices.

### Contribution to the literature

Our findings confirmed a previous study reporting that SIDS vulnerabilities make these countries susceptible to the influence of vested interests [31]. This makes the careful examination of policy alternatives in tobacco governance challenging. Consequently, government agencies were likely to rely on the readily available information provided by the tobacco industry or development partners. Prior research in tobacco control in PSIDS reported on the detrimental impact of capacity issues on policymaking and implementation [37, 74, 75]. Other studies focusing on food policy in PSIDS had similar results; they showed that limited human and financial capacity was a major barrier to the development of multisectoral food policies [34, 76–78]. Our study provided new depth to these insights by analysing how SIDS capacity constraints limited health sector actors' capability to implement and participate in policy and legislative processes that could ensure a level playing field if adequately employed.

Our findings confirmed previous reports stating that the lack of terms of engagement with the tobacco industry left public health government officials vulnerable to industry interference [79]. Other studies suggested that in those countries where tobacco industry interests were strongly present, such terms of engagement were less likely to be adopted due to the already existing industry interference [80, 81]. This could explain why in Fiji, no terms of engagement were in place; however, in Vanuatu, there were considerably fewer existing industry interests than in Fiji. The institutional weaknesses identified by this study provide an alternative explanation of why Vanuatu and so many other LMICs had failed to implement Art. 5.3 of FCTC [82].

The administrative process theory proved to provide a useful theoretical lens to analyse institutional conditions in tobacco governance. While a previous study on tobacco governance validated the claims of the administrative process theory by showcasing a good example [32], despite the original intentions, this paper provided a case where lacking institutional conditions contributed to limited success in protecting health interests in tobacco governance. However, the administrative process theory did not explain the gap between the formal processes of policymaking and their actual use in policymaking. The governance and development scholarship has helped to explain this phenomenon. The governance and development literature [83–91] recognised that LMICs often establish democratic institutions as the means to become more legitimate for the public and development partners, while in reality, the way the country is governed relies on old, informal mechanisms, usually dominated by a political elite. Levitsky and Murillo [90] call these parchment reforms when “*rules exist on parchment, but in practice, they do little to constrain actors or shape their expectations*”. Pritchett et al. [85] describe this as *"isomorphic mimicry, wherein the outward forms (appearances, structures) of functional states and organisations elsewhere are adopted to camouflage a persistent lack of function."* This could help understand why there was a gap between the rules and reality of policymaking in Fiji and Vanuatu; however, our study has expanded this understanding by explaining that it is not mere political calculations that were behind such façade but a complex interplay of SIDS vulnerabilities.

### Implications for policy

Despite the development partner support received by the governments of Fiji and Vanuatu, similarly to global trends [92], NCD prevention receives less financial assistance than other areas of health, and it does not target civil society. Funding CSOs to operate in this field in PSIDS would be an important step in strengthening health sector actors’ capacity to protect public health interests. Increasing the human, technical and financial capacity of MoH would support well-informed policy choices; it would provide adequate capacity to follow the policy process meaningfully, implement terms of engagement, and present more evidence on the harmful impact of supporting the tobacco industry. However, capacity building efforts must be sensitive to the cultural context of PSIDS [93]. These insights are aligned with prior suggestions to build capacity in PSIDS to strengthen tobacco control [37, 74, 75]. However, our findings imply that capacity building is unlikely to bring the desired outcomes without strengthening governance.

Our study suggests that strengthening performance management and accountability mechanisms would ensure that the carefully planned policy processes are followed, and it would improve the implementation of FCTC, which could result in the development of terms of engagement with the tobacco industry. This recommendation is aligned with CDoH scholars’ suggestions [94, 95], highlighting the need for robust accountability mechanisms to regulate harmful commodity industries; moreover, it corresponds to development scholars’ works [83, 86, 87], emphasising that government strengthening is essential to address the gap between the rules of policymaking and their implementation. Governance strengthening initiatives are frequently provided by development partners to LMICs, and there have been examples of such programmes in Fiji according to our data; however, the political elite had been selective on which recommendations of the development partners to follow, which led to our next insight.

The tightening of the regulation of the political parties and strengthening of the Parliament is necessary for PSIDS for the institutional conditions to ensure the protection of public interests. In both Fiji and Vanuatu, Parliamentary capacity needs to be improved to enable oversight over the government to facilitate the protection of public interests in governance and that the governance strengthening programmes bring the desired results. In Vanuatu, regulation of the political parties must be strengthened to decrease clientelism, patronage, and frequent rotation among high-level government officials. In Fiji, the transition to full democracy should be encouraged: the political elite needs to be persuaded to let go of its control, which had already started when the December 2022 elections in Fiji resulted in the change of government for the first time in 16 years [53].

### Limitations

This study was constrained by the funding and time restrictions of being a doctoral project. This resulted in several limitations. First, we have not had the resources to formally include local Fijian or ni-Vanuatu researchers. This limitation was mitigated by successfully requesting substantial guidance from government officials in MoH in Fiji and Vanuatu. Second, only two case studies were included in our study. This has a detrimental impact on the generalisability of the findings but allows the exploration of the institutional conditions shaping health sector actors’ capability to protect health interests in tobacco governance [96]. Third, recall bias might have weakened the data collected from interviewees [97]; this limitation was addressed by source triangulation [65].

## Conclusions

Health sector actors in Fiji and Vanuatu were not supported by institutional conditions that could help them protect public health interests in multisectoral governance to regulate CDoH originating from the tobacco industry. Institutional conditions in these states were shaped by SIDS vulnerabilities but could be improved by targeted capacity building, governance and political system strengthening. Changing the institutional conditions is a slow and lengthy process, but initiatives focusing on some relevant areas are already underway in Fiji and Vanuatu.

## Data Availability

The datasets used and/or analysed during the current study are available from the corresponding author on reasonable request.

## Abbreviations

AG: Attorney-General
BAT: British American Tobacco
CDoH: Commercial determinants of health
CSO: Civil society organisation
FCTC: Framework Convention for Tobacco Control
LMICs: Low- and middle-income countries
MoH: Ministry of Health
MP: Members of Parliament
NCD: Noncommunicable diseases
NGO: Non-governmental organisation
PM: Prime Minister
PSIDS: Pacific small island developing states
SIDS: Small island developing states
WHO: World Health Organization

## Footnotes

1 The MPOWER reports are published by the Tobacco Free Initiative, World Health Organization (WHO) Geneva, to track the progress of the implementation of tobacco control measures globally.

2 Second-in-command in a ministry in Fiji, the equivalent of DG in Vanuatu.
